# Clinical Presentations and Outcomes of Pediatric Rhegmatogenous Retinal Detachment: 11 Years’ Experience at a Tertiary Eye Center

**DOI:** 10.3390/medicina60091442

**Published:** 2024-09-03

**Authors:** Omar Alabbasi, Moustafa S. Magliyah, Hani Basher Albalawi, Heba M. Alsharif, Eman M. Alsharif, Ahmed Abu El-Asrar

**Affiliations:** 1 Vitreoretinal Division, King Khalid Eye Specialist Hospital, Riyadh 12329, Saudi Arabia; 2 Ophthalmology Department, Prince Mohammed Medical City, Sakaka 72345, Saudi Arabia; 3 Ophthalmology Division, Department of Surgery, Faculty of Medicine, University of Tabuk, Tabuk 47512, Saudi Arabia; 4 Anterior Segment Division, King Khalid Eye Specialist Hospital, Riyadh 12329, Saudi Arabia; 5 Ophthalmology Department, Prince Noura Bint Abdul Rahman University, Riyadh 11564, Saudi Arabia; 6 Ophthalmology Department, Faculty of Medicine, King Saud University, Riyadh 11451, Saudi Arabia

**Keywords:** outcome, pediatric rhegmatogenous retinal detachment, retinal detachment, RD, vitrectomy

## Abstract

*Background and Objectives*: Pediatric rhegmatogenous retinal detachment (RRD) represents a challenge for ophthalmologists and vitreoretinal surgeons. In this study, we aim to review the clinical features, and surgical and visual outcomes of pediatric RRD in a tertiary referral center. *Materials and Methods*: This is a retrospective study using the review of charts for all patients who presented to King Abdul-Aziz University Hospital, Riyadh, Saudi Arabia, from 2005 to 2016. This study included patients 18 years old or younger, had undergone surgical repairs for RRD, and were followed up for 1 year or more. *Results*: Eighty-nine eyes of 70 children who underwent surgical repair for RRD were included in the current retrospective study. A previous history of intraocular surgeries was present in 31.5%. The majority of cases had known ocular disease or syndromes (n = 58, 65.2%). The majority of eyes which had a primary surgical intervention achieved anatomical success (n = 88). Corneal complications accounted for the majority of complications following primary and secondary surgical interventions. Forty-two percent of the eyes had vision better than 20/200, while thirty-five percent had vision of hand motion or worse. *Conclusions*: In conclusion, despite the variability in causes of RRD in children, successful anatomical outcomes can be achieved with the proper surgical approach. Visual outcomes are less predictable due to other ocular complications.

## 1. Introduction

Retinal detachment (RD) is a sight-threatening condition. It is defined as a separation of the neurosensory retina (NSR) from the underlying retinal pigment epithelium (RPE) [1]. There are four main types of retinal detachments: exudative (ERD), tractional (TRD), rhegmatogenous (RRD), and combined traction–rhegmatogenous retinal detachment [2]. It is estimated that the incidence of RRD is 1 in 10,000 in the general population. If RRD happens in one eye, the risk is approximately 10% in the fellow eye [3]. In the pediatric population, the incidence of RD is lower, accounting for 2–6% of all retinal detachments [4,5]. Epidemiological and clinical data of previous studies are provided and summarized in Table 1.

Compared to adult retinal detachments, pediatric retinal detachments usually have poor prognoses. Factors which contribute to the poor prognoses include late presentations, other ocular complications, and high rates of proliferative vitreoretinopathy (PVR) [8,14]. Among pediatric patients, males were found to be more commonly present with RRD [19]. However, when cases of trauma were excluded, equal proportions between male and female detachments were found [17]. The presentation, diagnosis, and management of RD in the pediatric age group confer many challenges. Symptoms were reported only in 40–70% of children with RD [6,8,15]. Bilateral RDs and the development of second-eye detachments are seen in 30% of children who have developed RRD [8,16]. High-risk peripheral retinal pathologies could be found in 80–90% of eyes with RRD [4,6,8,9,15,20]. Myopia is a well-known cause of RRD in children, especially when associated with syndromic features or congenital anomalies [7,12,13]. It is well-observed that trauma, preceding ophthalmic procedures, myopia, and developmental and congenital anomalies pose significant risks for RRD in the pediatric population [15,17,18,19,21,22,23,24]. Idiopathic detachments with no clear reasons were frequently found in previous studies. Some examples of common congenital abnormalities linked to RRD are summarized in Table 2.

In this retrospective study, we aim to review all pediatric patients who presented with RRD at a tertiary referral eye center. In addition, we aim to analyze the epidemiological characteristics, causes, anatomical and visual outcomes of pediatric RRD and surgical interventions.

## 2. Materials and Methods

This is a retrospective study using the review of charts for all patients who presented at King Abdul-Aziz University Hospital, Riyadh, Saudi Arabia, from 2005 to 2016. The study was performed according to the tenets of the Declaration of Helsinki, and approved by the Institutional Review Board (IRB code KS-20140-018 and date 12 June 2016). This study included patients who were 18 years old or younger, had undergone surgical repairs for RRD and were followed up for 1 year or more. A data collection sheet was designed including patients’ age, sex, main complaints, duration of symptoms, previous ophthalmic history, previous ophthalmic surgeries, the affected eye, the family history of ocular diseases, visual acuity (VA) upon presentation and the last follow-up visit, intraocular pressure (IOP) on presentation, refractive errors, strabismus, lens and vitreous status, the extent of retinal detachments and macula, the presence of PVR, the type and location of retinal holes and breaks, and any peripheral retinal changes.

Postoperatively, complications and their managements were recorded. The final anatomical and visual outcomes were analyzed and evaluated in relation to the presenting symptoms, the surgical procedures, the complications, and the number of surgeries performed.

Statistical analysis:

The data were collected and stored in a spreadsheet using Microsoft Excel 2010 software. Data management and coding were then carried out in Excel. Data were analyzed using SPSS^®^ version 20.0 (IBM Inc., Chicago, IL, USA).

Descriptive analysis was carried out, where categorical variables were presented in the form of frequencies and percentages, and continuous variables in the form of mean (±standard deviation). The Chi2 test was used to test associated final visual acuity and recurrent retinal detachment.

## 3. Results

In total, 89 eyes of 70 children (19 bilateral and+ 51 unilateral) which had undergone surgical repairs for RRD were included. In total, 49 patients were boys (70%). The mean age on presentation was 10.1 ± 4.6 years (Table 3). The most common presenting symptom was decreased vision (or visual loss), which was reported in 79%, followed by strabismus in 11.2% and leukocoria in 2.2%.

### 3.1. Past Ocular and Systemic History

**Figure 1:**
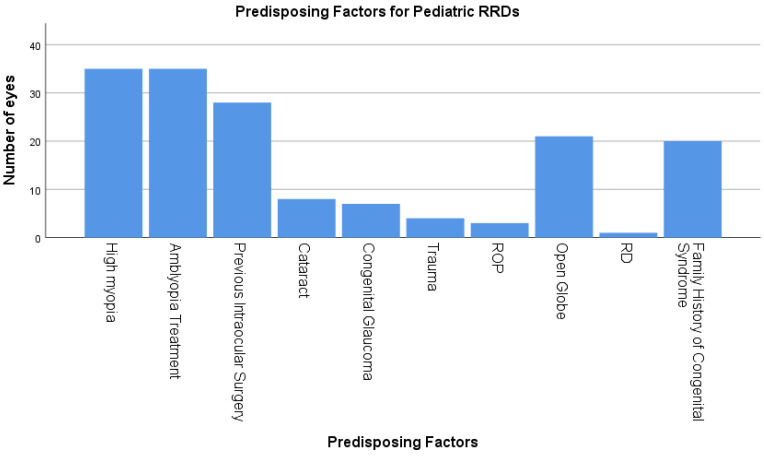
Predisposing factors for pediatric RRD: This is a bar chart of the most common predisposing factors for pediatric rhegmatogenous retinal detachments (RRD) including high myopia, amblyopia treatment, previous intraocular surgery, cataract, congenital glaucoma, trauma, retinopathy of prematurity, open globe, previous history of retinal detachment, and family history of a congenital syndrome.

A history of previous intraocular surgeries was documented in 31.5%. Indications for surgery included open globe in 21 (23.65%) eyes, cataract in 8 (9%) eyes, congenital glaucoma in 7 (7.9%) eyes, blunt trauma with intact globe in 4 (4.5%) eyes, retinopathy of prematurity in 3 (3.4%) eyes, and retinal detachment in 1 eye. The mean interval between the previous intraocular surgeries and the diagnosis of RRD was 1533 ± 1388 days. Figure 1 shows the predisposing factors for pediatric RRD.

The majority of the cases were found to have ocular diseases or syndromes (n = 58, 65.2%). The most common associated ocular causes of RDD were stickler syndrome (n = 22, 37.9%), then high myopia (n = 12, 20.7%), congenital cataract (n = 7, 12.1%), congenital glaucoma and retinopathy of prematurity (n = 5, 8.6%). Stickler syndrome was found to be the most common congenital syndrome associated with retinal detachment in our cohort of patients (n = 14, 73.7%) (Table 3).

### 3.2. Examination on Presentation

The visual acuity on presentation was hand motion or worse in (n = 33, 31.7%), while 30% of the patients had a visual acuity of counting fingers (n = 27) and only 9% of the patients had vision of ≥20/200 (n = 9).

**Figure 2:**
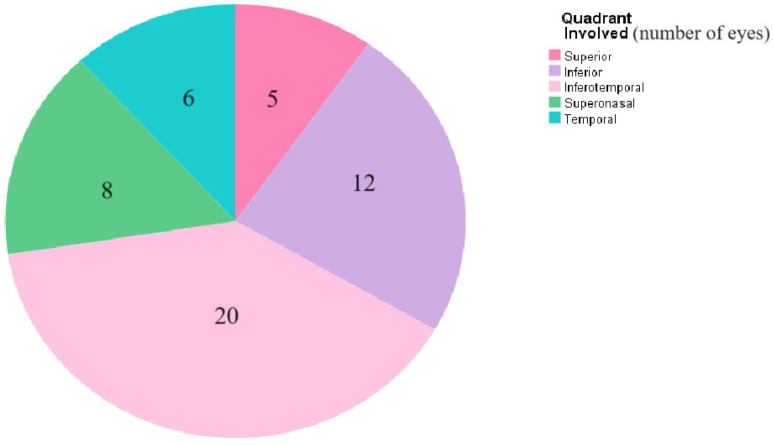
Retinal quadrants involved in the RRD. This is a pie chart showing the retinal quadrants involved in pediatric RRD which include superior, inferior, inferotemporal, superonasal and temporal quadrants of the retina.

The IOP was normal in the majority of the eyes (n = 66, 74.1%); four eyes (4.5%) had a high IOP and three eyes (3.4%) had a low IOP. More than sixty percent of the patients were orthotropic on the initial examination (n = 53), while 19 patients had an esotropia (ET) (21.3%) and 6 patients had an exotropia (XT) (6.7%). Forty-four percent of the patients were previously treated for amblyopia when they were initially diagnosed with retinal detachments (n = 40). Nystagmus was present on initial examination in only 11% of the patients (n = 10). More than two-thirds of the eyes had normal corneal examination and a quiet anterior chamber on initial examination (n = 76, 85.5%). The vast majority of the eyes were phakic (83.1%), and more than half of these had a clear lens (n = 46, 51.7%), while 29% had cataracts (n = 26). Furthermore, fifteen eyes were already operated on for cataract (16.8%), eight were aphakic (9%) and seven were pseudophakic (7.9%) at the time of presentation. The mean duration between cataract surgery and RRD diagnosis was 1903.8 ± 1407.4 days. Twenty-seven percent of eyes had optically empty vitreous (n = 24). Fundus examination on presentation revealed the presence of total RRD in more than half of the eyes (n = 51, 57.3%). The breaks were localizable in 57% of eyes (n = 51). The most common types of breaks were flap tears, giant tears, retinal dialyses and retinal holes. The majority of eyes had a single break (n = 24, 27%). The locations of retinal breaks are summarized in Figure 2. Signs of chronic RRD included thin retina (n = 54, 60.7%), demarcation lines (n = 43, 48.3%), and intraretinal cysts (n = 28, 31.5%). Fifty-eight percent of the eyes showed signs of proliferative vitreoretinopathy (PVR) on presentation (n = 52) and most eyes had PVR grade C (n = 34, 65%) followed by PVR grade B (n = 5, 9%).

Forty-two percent of the eyes had lattice degeneration (n = 38), while twenty-nine percent showed pigmentary changes (n = 26).

### 3.3. Primary and Secondary Surgical Intervention in Children with RRD

The majority of children with RRD had undergone pars plana vitrectomy plus encircling band as primary surgical interventions (n = 71, 79.8%), followed by pars plana lensectomy (n = 51, 57.3%). Peeling of posterior hyaloid and laser retinopexy were both performed in the vast majority of studied eyes (n = 87, 97.8%). Relaxing retinectomy was performed only in 27% (n = 24) of the studied eyes. The most common intraocular tamponading agent used was silicone oil (SO) in eighty-five percent (n = 76), while C3F8 was only used in four eyes.

The majority of cases which had undergone primary surgical interventions had successful surgery anatomically, with retinal flatting reaching up to ninety-eight percent (n = 88). In 70% of the studied eyes, the retina was attached on follow up, and silicone oil was later removed in 65 eyes (n = 58) after a mean duration of 311.4 ± 226.4 days. After silicon oil removal, 23% of eyes developed re-detachment (n = 6). The most common reason for re-detachment was PVR, which was seen in 92% of these eyes (n = 24).

In total, 19 eyes (21.3%) had undergone secondary surgical interventions after re-detachments. The most common type of intraocular tamponading agent used in reoperations was SO (n = 17, 19.1%). Seventeen cases had flat retina postoperatively, and two cases had end-stage funnel-shaped RRD.

### 3.4. Complications Postoperatively

**Figure 3:**
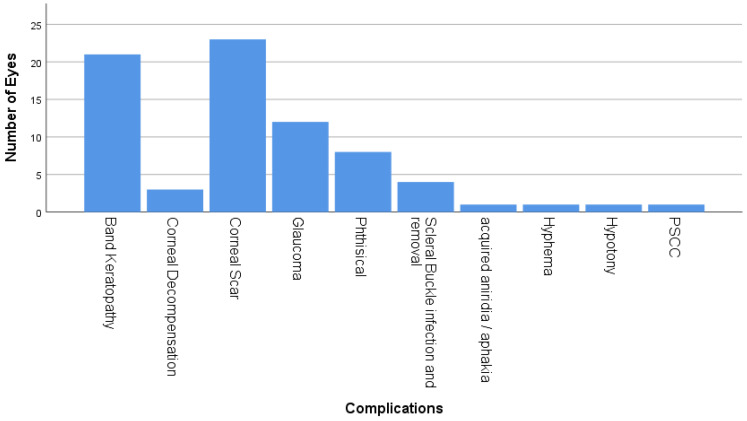
Postoperative ocular complications. This is a bar chart which shows the ocular complications of the pediatric RRD repair including band keratopathy, corneal decompensation, corneal scar, phthisis bulbi, scleral buckle infection and removal, acquired aniridia or aphakia, hyphema, hypotony and PSCC.

Corneal complications accounted for the majority of complications in children with RRD and included corneal scar (n = 22, 75%), band keratopathy (n = 21, 72%), and corneal decompensation (n = 3, 10.3%) Figure 3. Moreover, glaucoma was observed in 13 cases (n = 12). Four cases required trabeculectomy with mitomycin C, while two cases required Ahmed tube implant surgery. Eight of these eyes eventually developed pthisis bulbi. Other less common complications included scleral buckle infection, acquired aniridia, acquired aphakia, hyphema, hypotony and PSCC (Figure 3).

### 3.5. Final Visual Outcomes

On the last follow up, 42% of studied eyes had vision better than 20/200, while 35% had hand motion vision or worse. Ninety-five percent of studied eyes had attached retina on the last follow up and the mean follow-up duration was 33.2 ± 31.9 months (Table 4).

### 3.6. Risk Factors for RRD

Risk factors which had significantly predicted poor surgical outcomes were lens status on presentation (*p* = 0.017) and intraoperative complications (*p* < 0.001) (Table 5).

### 3.7. Factors Associated with Final Visual Acuity

There was a statistically significant relationship between the child’s age and VA of 20/200 or better (*p*-value < 0.001). Children above 5 years of age tended to have VA of 20/200 or better (Table 6). In addition, VA on presentation was an important determinant of the final visual outcome, where those who had VA ≥ 20/200 at presentation had better visual outcomes and the relationship was statistically significant (*p* < 0.001). The absence of PVR was associated with a better final visual outcome of ≥20/200 (*p* < 0.019).

### 3.8. Comparison of Initial and Final Visual Acuity:

A comparison between the initial and final VA in children who had undergone surgical interventions post RRD diagnosis showed that 23 of the cases had improved vision (34.8%), 32 of the cases remained the same (48.5%) and 11 cases had deteriorated vision (16.7%) (Table 7).

## 4. Discussion

In this study, the epidemiology, risk factors, presentation, surgical interventions and outcomes of management were analyzed.

A previous history of trauma was found in 27.7% of patients; 4.5% of patients had blunt trauma with an intact globe, while the majority had open globe injuries. Previous studies have shown similar rates (22% and 36%) in pediatric eyes with RRD [4,9,11,13,15]. Most other studies have found higher rates of trauma history (42% to 45%) [4,15,24,25,26]. This variability might be related to dissimilarity of the sample size, enrollment criteria and sex ratios. Most of the studied eyes had known ocular disease or syndromes (n = 58, 65.2%). The most common associated ocular causes of RDD were stickler syndrome, high myopia, congenital cataract, congenital glaucoma and ROP. Stickler syndrome was the most common congenital syndrome associated with RRD in the studied cohort, while only 7.9% of individuals with RRD were idiopathic. This rate is similar to what has been described by Wang et al., as they established that 4.7 percent of 296 eyes had no predisposing component [13]. Likewise, Gonzales et al. established the underlying conditions of retina in 98% of the 46 evaluated eyes [15].

Around one third of eyes with RRD have undergone prior ophthalmic surgeries. This observation is similar to the reported range of 34% to 60% in previous studies [9,15,25]. Nevertheless, Yokoyama et al. [11], Wang et al. [13], and Chang et al. [12] projected rates of 2 percent, 5 percent, and 6 percent, respectively, which are much lower. Like trauma, the gap in sample size, sex ratios and enrollment criteria act as an explanation of the differences between the previous studies and our study [11,13,15].

The presenting symptoms in children with RRD differ from adults. For instance, pediatric patients might be incapable of complaining about decreased vision or loss of vision, and a noteworthy count is incidentally discovered for chronic retinal detachments. Our study showed that the most common presenting symptoms were decreased vision in 79.8% of eyes and strabismus in 10 eyes (11.2%). Fivgas and Capone [23] established that poor vision was the most common presenting symptom. Wang et al. [13] studied 278 patients and discovered that the most common complaint was blurring of vision. Therefore, children with a detached retina might present late to ophthalmologic care. Late presentations can lead to a higher chance of PVR and fluid under the macula [14]. Worse visual acuity is seen in pediatric patients, with a higher percentage of macular involvement after greater delay, compared with the adult population [14]. Due to increased proliferation and cellular activity, in addition to delayed presentation, PVR is seen more in pediatric patients; PVR of grade C or more is seen in about 20–60 percent of these patients. Delay in diagnosis and referral occurs due to this uncommon disease having a lower clinical suspicion and because the peripheral retina is difficult to evaluate among children. Four quadrant detachments also have worse outcomes compared to three quadrant detachments [18].

The surgical repair of pediatric RRD involves the same surgical principles that guide the repair of adult detachment: relief of traction on the causative retinal defect or defects, reapproximation of the neurosensory retina to the retinal pigment epithelium (RPE), and creation of a retina to RPE adhesion. However, there are several key differences in the anatomy and function of the pediatric eye that demand careful consideration and change the relative advantages of different techniques. The majority of pediatric RRDs should be approached initially with a scleral buckle; a buckling element is chosen and sutured over the breaks. The drainage of the subretinal fluid is a controversial topic among vitreoretinal specialists [20,21]. Sparing the use of vitrectomy in pediatric patients is preferable for several reasons. Unlike adults who might have variable strength of posterior vitreous attachments, or even posterior vitreous detachments, a child’s posterior hyaloid often remains quite adherent to the retinal surface and is difficult to elevate anteriorly beyond certain levels in relation to the causative break. Vitreous that is left near the break might contract, leading to recurrent detachment. Also, pediatric vitreous is often more formed than adult vitreous. The surgeon can take advantage of cases with giant retinal tears through using the scleral buckle to relieve the tractions and using the vitreous itself to tamponade the retinal breaks, allowing for the resorption of subretinal fluid. Finally, vitrectomy is associated with higher rates of cataract development compared to scleral buckling. Because of amblyopia risk and loss of accommodation, pediatric cataracts are considerably more morbid than adult cataracts. Thus, scleral buckling is much more preferable in pediatric RRDs to minimize the risk of cataract development. Prior to the routine use of vitrectomy, scleral buckling alone was successful for the repair of 70–80% of pediatric RRDs [4,14,18,20,21]. RRDs with extensive PVR can even be successfully addressed with sclera buckling. In the case series by Akabane et al., the authors report the successful repair of 7/7 retinal detachments attempted with scleral buckling alone in cases with at least PVR grade C [7]. However, pars plana vitrectomy (PPV) is growing in popularity for the treatment of primary rhegmatogenous retinal detachment [22]. Indications and long-term results of PPV in children were comparable with those in adults. By far the most frequent indications for vitrectomy were injuries and their complications. In other pathologies, scleral buckling is a reasonable management approach.

The final rate of retinal reattachment of 95.5% is relatively close to the 72% to 96% that the previous literature reported [10,25,27]. There was an improvement in vision to 20/200 or better in 42% of eyes. There are similarities with previous studies of Akabane et al. [7], Soheilian et al. [17], and Wang et al. [13].

Significant predictors of primary surgical failure for RRD included lens status on presentation (*p*-value 0.017) and intraoperative complications (*p*-value < 0.001). Positive final VA predictors on univariate analysis were visual acuity of ≥20/200 at presentation (*p*-value < 0.001), and children aged above 5 years who tended to have a VA of ≥20/200. In addition, the absence of PVR was found to be a good prognostic factor and correlated to a better final visual outcome of ≥20/200 (*p*-value < 0.019). Moreover, there was a considerable correlation between the final visual outcome and phthisis bulbi as a postoperative complication, where in the absence of phthisis bulbi, patients tended to have a better VA of ≥20/200 (*p*-value < 0.001).

Tasman and Winslow [4] found that PVR and chronic RRD were associated with poor surgical outcomes. Yokoyama et al. [11] found associations between poor final outcomes and preoperative PVR, and initial poor VA. Weinberg et al. [9] clarified that poor outcome predictors were poor VA on presentation, the need for PPV, macular involvement, PVR grade C, and SO utilization. Wang et al. [13] reported that poor surgical outcomes are related to prior exposure to an intraocular surgery, congenital anomalies, a PVR of grade C, total RRD, macula off and SO utilization. Gonzales et al. [15] reported that poor outcomes were associated with younger age, greater extent of RRD, poor initial vision, along with PVR of grade C or lower. Chang et al. [12] carried out the analysis of logistic regression and outlined that macular involvement, PVR and non-myopic RRD are risk factors for poor surgical outcomes.

Anatomical success rates and visual outcomes were found to be the lowest among the younger age groups who had lower anatomical success rates as well [13]. These patients are also more likely to undergo vitrectomy and lensectomy [13]. In addition, postoperative problems peculiar to pediatric patients include higher intraocular cellular activity and difficulty in maintaining a posture. Therefore, the possibility of recurrent detachments and PVR after surgery are higher in patients requiring vitrectomy than in the patients requiring buckling surgery [18]. The poorest outcomes after repair tend to be in patients with congenital abnormalities, trauma, and prior surgeries [8,11,12], with only 22–44% success rate for the patients with prior ocular surgeries [8,12]. Other risk factors for poor outcomes are macular involvement, the presence of PVR, the presence of a giant retinal tear, and inability to determine preoperative acuity [9,13,16,17].

## 5. Conclusions

Pediatric rhegmatogenous retinal detachment is considered challenging for ophthalmologists and vitreoretinal surgeons in terms of clinical presentations, pediatric examinations, surgical interventions, risk of amblyopia as well as worse complications compared to the adult age group. Despite this, successful anatomical outcomes can be achieved with the proper surgical approach. Scleral buckling might provide more favorable long-term outcomes, especially in non-trauma cases.

## Figures and Tables

**Table 1:** Demographic, clinical data, and interventions performed in the included studies.

First Author	Eyes/ Patients (*n*)	Mean Age (a); Sex (M/F,%)	Traumatic/Myopic/Congenital-Developmental/Other (%)	No. of Interventions (Mean)	Primary S.B/Vitrectomy (%)	Total Vitrectomy (%)	Combined S.B-Vitrectomy (%)	Use of Silicone Oil (%)
Haring [6] 1998	33/31	15; 54/46	24/42/0/NR	1.2	100/0	3	0	NR
Akabane [7] 2001	32/28	12.8; 65/35	22/38/16/24	NR	78/22	31	0	NR
Fivgas [8] 2001	29/27	9.6; 70/30	25–60/3/45/51	2.2	28/72	92	0	72
Weinberg [9] 2003	39/34	9.2; 79/21	36/NR/53/15	1.6	41/13	67	46	23
Sarrazin [10] 2007	37/36	11; 86/14	100/0/0/0	74% > 1; Range 1–4	NR	NR	100	54
Yokoyama [11] 2004	55/49	12; 86/14	27 /25(>4 D)/15/33	1.2	76/24	38	0	NR
Chang [12] 2005	152/146	13.1; 70/30	33/37/12/17	1.5	61/39	44	see vitrectomy	32
Wang [13] 2014	296/278	14.6; 74/26	31/79; 38 (>6 D)/17/0	1.34	76/5	55	19	9
Rumelt [14] 2007	144/127 (115 RRD)	10.8; 73/27	42/14; 3 (>6 D)/36/22	67% 1; 19% 2; 14% 3 to 8	NR	NR	NR	NR
Gonzales [15] 2008	46/45	9.8; 71/29	43/17 (>4 D)/35/11	50% > 1 (range 1–3)	26/44	74	30	57
Wadhwa [16] 2008	230/216	11.1; 82/18	34/14/24/28	1.98	37/0 (see association)	69	63	69
Soheilian [17] 2009	127/108	12.1; 81/19	43/9 (8 > 8 D)/38 (some)/2	1.55	31/0 (see association)	75	63	65
Oono [18] 2012	48/44	12.3; 88/12	38 /44 (>3 D); 25 (alone)/10/27	1.46	77/23	NR	0	19

NR: Not reported; D: Dioptre; S.B: Scleral Buckle.

**Table 2:** Genetic and clinical features of inherited disorders which are associated with a higher risk of pediatric RRD.

Marfan Syndrome	Dominant Mutations in the Fibrillin-1	If there’s RD in One Eye Other Eye will be Affected in around 70%
Wagner disease and erosive vitreoretinopathy	Autosomal dominant vitreoretinopathy caused by mutations in the VCAN gene 3.	Myopia, night blindness, presenile cataract, retinal detachment, optically empty central vitreous, peripheral strands and veils, chorioretinal atrophy, adult-onset glaucoma and ocular hypertension have been reported.
Stickler syndrome	Autosomal dominant. Mutations reported in COL2A1, COL11A1, COL11A2, COL9A1, and COL9A2 genes.	Most common ophthalmic complication of stickler in RD accounts for around 70%.
Knobloch syndrome	Autosomal recessive disorder caused by mutation in the COL18A1 gene.	Characterized by high myopia ranging from (−10 to −20 D), vitreoretinal degeneration with retinal detachment, and occipital encephalocele.
X-linked juvenile retinoschisis	Affects males early in life; mutation of RS1 gene.	It is characterized by radial streaks in macula secondary to foveal schisis as well as splitting of the peripheral temporal retina. RD can occur only after break of both inner and outer retinal layers.
Choroidal coloboma	Inherited or can occur sporadically. Can happen secondary to failure of closure of the embryonic fissure.	RRD secondary to congenital colobomas has been reported to reach up to 8% in children.
Retinal vascular disease	Retinal vascular disease is a usual cause of tractional retinal detachment but rhegmatogenous retinal detachments (RRDs) can also occur as late sequelae of tractional retinal detachments.	Retinal vascular diseases can cause RRD including regressed retinopathy of prematurity (ROP), familial exudative vitreoretinopathy (FEVR), persistent fetal vasculature, incontinentia pigmenti, uveitis, and sickle cell retinopathy.

**Table 3:** Descriptive analysis of demographics and relevant history.

Characteristic	N (%)
Age in years (mean ± SD [range], median)	10.1 ± 4.6 [2–18], 10.0
Gender	
Male	49 (70.0)
Female	21 (30.0)
Laterality	
Right Eye	31 (44.3)
Left eye	20 (28.6)
Both eyes	19 (27.1)
History of Previous Ocular Surgeries	
Cataract extraction + intraocular lens implantation	3 (3.4)
Trabeculectomy	5 (5.6)
Ahmed valve implant	1 (1.1)
Lens aspiration + anterior vitrectomy	7 (4.2)
Deep sclerotomy	1 (1.1)
PPV + PPL + silicone oil	2 (2.2)
Primary repair then, PPV, PPL removal of an intraocular foreign body	4 (4.5)
Primary repair + penetrating keratoplasty later on	1 (1.1)
Interval between the previous surgery and the diagnosis of RRD in days (mean ± SD [range], median)	1533 ± 1388 [2–4280], 1265
Predisposing Ocular Pathologies	58 (65.2)
Stickler syndrome	22 (37.9)
Congenital cataract	7 (12.1)
High myopia	12 (20.7)
Congenital glaucoma	5 (8.6)
Retinopathy of prematurity	5 (8.6)
Primary congenital glaucoma	2 (3.4)
Anterior uveitis	1 (1.1)
Optic nerve coloboma	1 (1.1)
Familial exudative vitreoretinopathy	1 (1.1)
Nanophthalmos	1 (1.1)
Paras planitis	1 (1.1)
Sickle cell anemia	1 (1.1)
Family History of Congenital Syndromes	19 (27.1)
Stickler	14 (73.7)
Down syndrome	2 (10.6)
Familial exudative vitreoretinopathy	1 (5.3)
Joubert syndrome	1 (5.3)
Sickle cell anemia	1 (5.3)

PPV: pars plana vitrectomy, PPL: pars plana lensectomy.

**Table 4:** Outcomes of pediatric RRD management.

Final VA	
≥20/200	37 (41.6)
CF	15 (16.9)
NLP-HM	31 (34.8)
Final IOP	
Low	1 (1.1)
Normal	68 (76.4)
High	3 (3.4)
Attached Retina	85 (95.5)
Follow-up duration in months (mean ± SD [range], median)	33.7 ± 31.8 [2–144], 24.0

CF: counting fingers, NLP: no light perception, HM: hand motion, IOP: intraocular pressure.

**Table 5:** Risk factors for RRD.

Variable	Failed Primary Surgery—Yes (n = 26) n (%)	*p* Value
Gender		
Male (n = 67)	22 (32.8)	0.190
Female (n = 22)	4 (18.2)	
Age in years		
≤5 (n = 21)	6 (28.6)	0.941
>5 (n = 68)	20 (29.4)	
Laterality		
Unilateral (n = 51)	13 (25.5)	0.371
Bilateral (n = 38)	13 (34.2)	
Trauma		
Yes (n = 23)	7 (30.4)	0.881
No (n = 66)	19 (28.8)	
Underwent primary repair		
Yes (n = 5)	1 (20.0)	0.641
No (n = 84)	25 (29.8)	
Past ocular history		
Congenital cataract		
Yes (n = 9)	3 (33.3)	0.774
No (n = 80)	23 (28.8)	
Congenital glaucoma		
Yes (n = 7)	3 (42.9)	0.408
No (n = 82)	23 (28.0)	
Visual acuity at presentation (20 missing)		
HM-LP/NLP (n = 33)	12 (36.4)	0.347
CF (n = 27)	6 (22.2)	
≥20/200 (n = 9)	4 (44.4)	
Proliferative vitreoretinopathy on presentation		
Yes (n = 52)	18 (34.6)	0.184
No (n = 37)	8 (21.6)	
Macula		
On (n = 5)	1 (20.0)	0.641
Off (n = 84)	25 (29.8)	
Lens status at presentation		
Phakic (n = 48)	8 (16.7)	0.017 *
	
Cataract (n = 26)	12 (46.2)	
Aphakia/pseudophakia (n = 15)	6 (40.0)	
Preoperative IOP		
Normal (n = 84)	24 (28.6)	0.585
High (n = 5)	2 (40.0)	
Surgical procedures		
Pars plana lens aspiration		
Yes (n = 51)	19 (37.3)	0.053
No (n = 38)	7 (18.4)	
Encircling band		
Yes (n = 71)	22 (31.0)	0.465
No (n = 18)	4 (22.2)	
Peeling of posterior hyaloid		
Yes (n = 28)	6 (21.4)	0.274
No (n = 61)	20 (32.8)	

* Statistically significant at 5% level of significance. CF: counting fingers, NLP: no light perception, LP: light perception, HM: hand motion.

**Table 6:** Factors associated with good vision at final follow up.

Variable	Good Vision ≥20/200 Yes (n = 31) n (%)	*p* Value
Gender		
Male (n = 63)	27 (42.9)	0.066
Female (n = 20)	4 (20.0)	
Age in years		
≤5 (n = 18)	1 (5.6)	0.002 *
>5 (n = 65)	30 (46.2)	
Laterality		
Unilateral (n = 49)	16 (32.7)	0.288
Bilateral (n = 34)	15 (44.1)	
Trauma		
Yes (n = 23)	9 (39.1)	0.835
No (n = 60)	22 (36.7)	
Underwent primary repair		
Yes (n = 5)	3 (60.0)	0.280
No (n = 78)	28 (35.9)	
Past ocular history		
Congenital cataract		
Yes (n = 9)	2 (22.2)	0.320
No (n = 74)	29 (39.2)	
Congenital glaucoma		
Yes (n = 6)	1 (16.7)	0.277
No (n = 77)	30 (39.0)	
Visual acuity at presentation (17 missing)		
HM-LP/NLP (n = 31)	9 (29.0)	0.031 *
CF (n = 26)	10 (38.5)	
≥20/200 (n = 9)	7 (77.8)	
Proliferative vitreoretinopathy on presentation		
Yes (n = 48)	12 (25.0)	0.006 *
No (n = 35)	19 (54.3)	
Macula		
On (n = 5)	3 (60.0)	0.280
Off (n = 78)	28 (35.9)	
Lens status at presentation		
Phakic (n = 46)	23 (50.0)	0.025 *
Cataract (n = 24)	6 (25.0)	
Aphakia/pseudophakia (n = 13)	2 (15.4)	
Preoperative IOP		
Normal (n = 78)	30 (38.5)	0.408
High (n = 5)	1 (20.0)	
Surgical procedures		
PPL lens aspiration		
Yes (n = 48)	16 (33.3)	0.376
No (n = 35)	15 (42.9)	
Encircling band		
Yes (n = 67)	25 (37.3)	0.989
No (n = 16)	6 (37.5)	
Peeling of posterior hyaloid		
Yes (n = 25)	13 (52.0)	0.070
No (n = 58)	18 (31.0)	
Tamponade used (n = 80)		
Gas (n = 4)	4 (100)	0.006 *
Silicone oil (n = 76)	27 (37.4)	

* Statistically significant at 5% level of significance. CF: counting fingers, NLP: no light perception, LP: light perception, HM: hand motion.

**Table 7:** Comparison of initial and final visual acuity.

Final Visual Acuity	Visual Acuity at Presentation			Total
	NLP/LP-HM	CF	≥20/200	
≥20/200	9	10	7	26 (39.4%)
CF	4	7	1	12 (18.2%)
NLP/LP-HM	18	9	1	28 (42.4%)
Total	31 (47.0%)	26 (39.4%)	9 (13.6%)	66 (100%)

Above the diagonal line, visual acuity had improved (23 (34.8%)), along the diagonal line, visual acuity remained the same (32 (48.5%)), and below the diagonal line, visual acuity deteriorated (11 (16.7%)). CF: counting fingers, NLP: no light perception, LP: light perception, HM: hand motion.

## Data Availability

The work was conducted at King Abdulaziz University Hospital (KAUH) and all related data are available at KAUH.
